# The first report of complete mitogenomes of two endangered species of genus *Propomacrus* (Coleoptera: Scarabaeidae: Euchirinae) and phylogenetic implications

**DOI:** 10.1371/journal.pone.0310559

**Published:** 2024-09-18

**Authors:** Chuanhui Yi, Xu Shu, Lingmin Wang, Jing Yin, Youhui Wang, Yuchen Wang, Honghui Zhang, Qiuju He, Min Zhao

**Affiliations:** 1 Yunnan Institute of Biological Diversity, Southwest Forestry University, Kunming, Yunnan Province, P.R. China; 2 Department of Forest Conservation, College of Biodiversity Conservation, Southwest Forestry University, Kunming, Yunnan Province, P.R. China; 3 Division of Resource Insects, Institute of Highland Forest Science, Chinese Academy of Forestry, Kunming, Yunnan Province, P.R. China; 4 Key Laboratory of Breeding and Utilization of Resource Insects, National Forestry and Grassland Administration, Kunming, Yunnan Province, P.R. China; Central University of Punjab, INDIA

## Abstract

To understand the mitochondrial genome structure of two endangered and long-armed scarab beetles, *Propomacrus davidi* and *Propomacrus bimucronatus*, their complete mitogenomes were sequenced for the first time in this study. The complete mitogenomes of *P*. *davidi* and *P*. *bimucronatus* were 18, 042 bp and 18, 104 bp in length, respectively. The gene orders of their mitogenomes were highly consistent with other Coleopteran species, and the typical ATN was used as the start codon in most protein coding genes. The incomplete stop codon T was used in *cox1*, *cox2*, and *nad5*, and TAN was used as a complete stop codon in most protein coding genes. All predicted tRNAs could form a typical cloverleaf secondary structure, except that trnS1 lacked the dihydrouridine arm. Based on the maximum likelihood and the Bayesian inference methods, phylogenetic trees of 50 species were reconstructed. The results showed that *P*. *davidi*, *P*. *bimucronatus*, *Cheirotonus jansoni* and *Cheirotonus gestroi* clustered in the same branch, and were the most closely related. The results supported that subfamily Euchirinae is a monophyletic group of Scarabaeidae, which was consistent with the morphological classification. These molecular data enriched the complete mitogenome database of Euchirinae, and improved our understanding of the phylogenetic relationship and evolutionary characteristics of these two endangered species.

## Introduction

The genus *Propomacrus* Newman, 1837 belongs to the subfamily Euchirinae in the order Coleoptera. It only includes three species, *Propomacrus bimucronatus* (Pallas, 1781), *P*. *davidi* Deyrolle, 1874 and *P*. *muramotoae* Fujioka, 2007. *Propomacrus cypriacus* Alexis & Makris, 2002 has been a subspecies of *P*. *bimucronatus* by genetic evidence [1]. The species of *Propomacrus* are giant beetles, and mainly found in the alpine regions of Asia, the Near East and southeastern Europe [1, 2], with high habitat requirements, sensitivity to environmental changes, poor adaptability, weak reproductive capacity, and long larval stage [3–5]. In recent years, due to the intensification of human activity intervention and serious damage to forests, the distribution areas of *Propomacrus* have declined sharply and some species have become endangered. *P*. *davidi* is endemic to China and only distributed in Jiangxi and Fujian provinces. It includes two subspecies, *Propomacrus davidi davidi* Deyrolle, 1874 and *Propomacrus davidi fujianensis* Wu, 2008 [6–9]. The beetle listed as one of the Grade II Key Protected Wild Animals based on the endangered status in China (https://www.forestry.gov.cn/main/5461/20210205/122418860831352.html). *P*. *bimucronatus* shows an intermittent distribution in Cyprus, Greece, Iran, Israel, Syria, Turkey, and the southern and central Balkans [1, 3, 10]. It was listed as Near Threatened (NT) in the European Red List of Saproxylic Beetles [11]. The adults of *P*. *davidi* and *P*. *bimucronatus* feed on the sap of trees and rotten fruits. The larvae feed on rotten wood and humus soil, which can accelerate the decomposition of dead wood and litter in forests [1]. They play an important role in the material cycle of forest ecosystems and have considerable ecological value. Meanwhile, the two species are famous ornamental insects because of their peculiar appearance and good ornamental value [12, 13]. Recent studies on the two beetles *P*. *davidi* and *P*. *bimucronatus* has mainly focused on morphological classification, biology, and ecology [2, 3, 9, 10, 14], only a few studies involved molecular biology [2, 8], and only *cox1* gene and *16S* of the two species were sequenced [1].

The family Scarabaeidae, within the order Coleoptera, comprises over 30,000 described species, with over 21 subfamilies [15]. These subfamilies exhibit distinct divisions in their primary feeding ecologies, broadly categorized into saprophagous and phytophagous lineages [15, 16]. The former mainly feed on decaying matter, animal dung, etc., including the subfamilies Scarabaeinae and Aphodiinae, while the latter typically consume plant fruits, flowers, pollen, tree sap, wood (including deadwood), leaves, etc. This group includes the four main subfamilies Melolonthinae, Rutelinae, Cetoniinae, and Dynastinae, as well as some less numerous subfamilies such as Euchirinae.

Euchirinae, a subfamily of Scarabaeidae, is an under-studied group with uncertain taxonomic status [3, 10, 17, 18], which includes three genera and 15 species and seven subspecies worldwide, namely 10 species and one subspecies of *Cheirotonus* Hope, 1841, two species and one subspecies of *Euchirus* Burmeister and Schaum, 1840, and three species and two subspecies of *Propomacrus* Newman, 1837. The phylogenetic study of Euchirinae has been conducted poorly, and it has been mainly based on morphological characteristics, rarely using molecular characteristics. Although its monophyly has been confirmed, the classification at the subfamily level remains uncertain [3, 19, 20]. Some scholars believe that Euchirinae is a subfamily of the family Scarabaeidae [10, 21, 22], but the evidence is needs to be more substantial [18, 23]. The subfamily is included in the subfamily Melolonthinae as tribe Euchirini sometimes [3] or in a clade composed of several representatives of Melolonthinae, Rutelinae, Dynastinae, as well as Cetoniinae [6, 23], or an independent family Euchiridae [1, 20]. Therefore, it is very important to develop reliable markers to reconstruct the phylogenetic relationship of the subfamily Euchirinae.

As a complete organelle genome, the mitochondrial genome (mitogenome) has the characteristics of maternal inheritance, small molecular weight, fast evolution rate and low recombination level [24]. Thus, it is a good marker for studying the phylogenetic relationships between species and genera. Therefore, complete mitogenomes have been used as a common molecular marker in insect systematics studies [25, 26]. The mitogenome of insect is a closed circular and double-stranded DNA molecule of 14–20 kb in length [27, 28]. As of May 2024, the number of mitochondrial genomes of Scarabaeidae insects stored in GenBank has exceeded 100, primarily from seven subfamilies: Scarabaeinae, Melolonthinae, Rutelinae, Cetoniinae, Aphodiinae, Dynastinae, and Euchirinae. Mitochondrial gene characteristics of over 30 Scarabaeidae species have been described and used for phylogenetic analysis [6, 29]. Just as mitochondrial genomes of coleopteran insects tend to be conservative [30], with the exception of some species in the subfamilies Dynastinae and Scarabaeinae that show gene rearrangements, the mitochondrial genome structure, base composition, codon usage, and gene arrangement of other Scarabaeidae species exhibit high similarity to other coleopterans [31, 32]. However, only three species of Euchirinae, *Euchirus longimanus*, *Cheirotonus gestroi*, and *C*. *jansoni*, are recorded in GenBank [6, 20, 29]. More complete mitogenome data of the Euchirinae species are needed for phylogenetic analysis.

In this study, the complete mitochondrial genomes of two species of genus *Propomacrus* (Coleoptera: Scarabaeidae: Euchirinae) were sequenced and analyzed for the first time. Combining these data with other available mitogenomes from GenBank, the phylogenetic relationships of Scarabaeoidea were reconstructed, which provides new insights into the relationships among Euchirinae and the other major subfamilies or families. These findings will be useful for better conserving endangered groups in the future.

## Materials and methods

### Specimen materials

The adult specimens of *P*. *davidi davidi* and *P*. *bimucronatus* were obtained from beetle breeding enthusiast in Kunming, Yunnan Province, China. Their ancestors were roughly mapped to the Longhushan Geopark in Jiangxi, China and Izmir, Turkey, respectively. They were reared from one instar in October 2019 and emerged in August 2020. Adult samples were stored in absolute ethanol and kept in the herbarium of Southwest Forestry University.

### Mitochondrial genome sequencing

The total DNA of the samples was extracted from the muscles of the adult’s thorax using a Magnetic Animal Tissue Genomic DNA Kit (DP341) (TIANGEN, Beijing, China). After the quality detection by 1.0% agarose gel electrophoresis, the DNA was sent to Baiai Gene Information Technology Co., Ltd. (Xi’an, China) for sequencing. Whole Genome Shotgun (WGS) was used to construct the library, and next generation sequencing was used to perform Paired-end (PE) sequencing on the Illumina sequencing platform.

### Sequence splicing and annotation

High quality next-generation sequencing data were assembled using SPAdes v3.9.0 [33] and contig and scaffold sequences were constructed. After comparing the sequence with the NT library on NCBI BLAST (BLAST v2.2.31+), the mitochondrial sequence of the splicing results was picked out. Combined with the reference sequences *C*. *jansoni* (GenBank ID: NC_023246) [34] and *C*. *gestroi* (NC_046890) [20], the mitochondrial sequences of splicing results were integrated. Collinearity analysis was performed using mummer v3.1 [35] to determine the positional relationship between contigs and fill the gap between contigs. The results were corrected using Pilon v1.18 software [36] to obtain the final mitochondrial sequence. The complete mitogenome sequence was uploaded to the MITOS website (http://mitos2.bioinf.uni-leipzig.de/index.py) for functional annotation [37]. The secondary structure of tRNA was predicted by tRNA Scan-SE [38] and MITOS web [37], and it was manually corrected by the SVG online editor. The complete mitogenome map was drawn using OGDRAW v1.3.1 [39].

### Phylogenetic analyses

Based on the mitogenome sequence of 48 species from four families of Scarabaeoidea, published in the NCBI database, two species from Staphylinidae and Byturidae were selected as outgroups (Table 1). The species selected from the superfamily Scarabaeoidea include 25 species from six subfamilies of the family Scarabaeidae, including Cetoniinae, Dynastinae, Rutelinae, Melolonthinae, Euchirinae, and Scarabaeinae, as well as two species from the subfamilies Hybosorinae and Ceratocanthinae of the family Hybosoridae, two species from the subfamily Passalinae of the family Passalidae, and 19 species from the subfamily Lucaninae of the family Lucanidae. The selected species were used to construct a phylogenetic tree with the two species and outgroups. PhyloSuite v1.2.2 aligned the complete mitochondrial genome sequence [40]. IQ-TREE v1.6.12 [41] and MrBayes v3.2.7a [42] were used to construct phylogenetic trees of 13 PCGs based on the maximum likelihood (ML) and Bayesian inference (BI) methods.

**Table 1:** Taxa used for phylogenetic analysis in this study.

Superfamily	Family	Subfamily	Species	GenBank ID	Length (bp)	Reference
Scarabaeoidea	Scarabaeidae	Cetoniinae	*Glycyphana fulvistemma*	NC_063847	16,701	N/A
			*Gametis jucunda*	NC_063846	17,980	N/A
			*Coenochilus striatus*	NC_065313	15,480	N/A
			*Trichius succinctus*	NC_063849	18,358	N/A
			*Dicronorhina derbyana*	OK484300	16,609	[31]
			*Eudicella smithii*	OK484302	16,712	[31]
			*Eudicella quadrimaculata*	OK484299	16,690	[31]
			*Protaetia brevitarsis*	NC_023453	20,319	[43]
			*Osmoderma opicum*	NC_030778	15,341	[44]
			*Mecynorhina polyphemus*	OK484305	16,422	[31]
		Dynastinae	*Eupatorus gracilicornis*	NC_065036	18,391	N/A
			*Eupatorus hardwickei*	ON817147	18,494	N/A
			*Eupatorus sukkiti*	ON756028	18,445	N/A
			*Megasoma mars*	OK484311	16,983	[31]
			*Trichogomphus mongol*	OM161966	17,377	N/A
		Rutelinae	*Anomala russiventris*	NC_065310	15,601	[45]
			*Popillia japonica*	NC_038115	16,541	[6]
		Melolonthinae	*Sophrops subrugatus*	NC_065314	16,409	[45]
			*Rhopaea magnicornis*	NC_013252	17,522	[46]
		Euchirinae	*Cheirotonus gestroi*	MN893347	16,899	[20]
			*Cheirotonus jansoni*	NC_023246	17,249	[34]
			*Propomacrus davidi*	NC_070351	18,042	This study
			*Propomacrus bimucronatus*	NC_070352	18,104	This study
		Scarabaeinae	*Dichotomius schiffleri*	NC_039689	14,802	[47]
			*Copris tripartitus*	NC_045923	15,457	[32]
	Hybosoridae	Hybosorinae	*Hybosorus* sp.	JX412807	12,639	N/A
		Ceratocanthinae	*Ceratocanthus* sp.	JX412772	12,579	N/A
	Passalidae	Passalinae	*Leptaulax koreanus*	NC_044848	18,730	[48]
			*Ophrygonius* sp.	NC_060602	16,195	N/A
	Lucanidae	Lucaninae	*Lucanus cervus*	MN_580549	20,109	[49]
			*Lucanus kanoi kanoi*	MK238348	17,991	N/A
			*Lucanus chengyuani*	MK878514	16,926	[50]
			*Lucanus fortunei*	NC_044961	16,591	[51]
			*Lucanus maculifemoratus taiwanus*	MK250907	21,174	N/A
			*Macrodorcas seguyi*	NC_038212	17,955	[52]
			*Serrognathus platymelus*	NC_044096	16,790	[53]
			*Dorcus hansi*	NC_043928	18,130	N/A
			*Prosopocoilus astacoides*	NC_050851	17,746	N/A
			*Prosopocoilus bulbosus*	MK134566	16,642	N/A
			*Prosopocoilus laterotarsus*	NC_065362	17,333	[54]
			*Prosopocoilus gracilis*	NC_027580	16,736	[55]
			*Rhaetus westwoodii*	MG159815	18,131	[56]
			*Kirchnerius guangxii*	NC_048957	14,562	[53]
			*Nigidius miwai*	NC_063663	18,462	N/A
			*Nigidius sinicus*	NC_065111	18,394	N/A
			*Prismognathus prossi*	NC_044962	15,984	[51]
			*Figulus binodulus*	NC_045102	16,261	[57]
			*Odontolabis cuvera fallaciosa*	MF908524	19,614	[58]
Staphylinoidea	Staphylinidae	Staphylininae	*Creophilus maxillosus*	NC_061687	17,395	N/A
Cucujoidea	Byturidae	-	*Byturus ochraceus*	NC_036267	15,143	N/A

Note:“-” stands for not available; “N/A” means unpublished.

## Results and discussion

### Mitogenome composition

**Fig 1:**
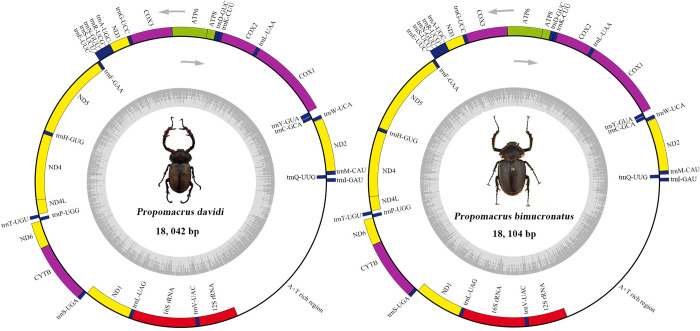
Circular maps of the mitogenome of *Propomacrus davidi* and *P*. *bimucronatus*. The direction of gene transcription is shown as an arrow. Dark blue represents transfer RNA (tRNA), yellow represents nicotinamide adenine dinucleotide (*nadh*), purple represents cytochrome c oxidase (*cox*) and cytochrome b (*cytb*), green represents adenosine triphosphate ATP synthase, and red represents ribosomal RNA (rRNA).

The complete mitogenome lengths of *P*. *davidi* and *P*. *bimucronatus* were 18,042 bp and 18,104 bp, respectively, which were longer than the 16, 899 bp of *C*. *gestroi* in the same subfamily [20]. Most metazoan mitogenomes are approximately 16 kb in size [59, 60]. Longer mitochondrial genes may be characteristic of *Propomacrus* species, which is closely related to the length of control region sequence. The control regions of *P*. *davidi* and *P*. *bimucronatus* are 765–1,202 bp longer than in *C*. *gestroi* and *C*. *jansoni* in same subfamily. The A + T content was 69.49% for *P*. *davidi*, and 67.91% for *P*. *bimucronatus*, with high AT-skew. The gene composition and sequence were consistent with those of ancestral insects, including 13 protein coding genes (PCGs), 22 tRNAs, two rRNAs, and a control region (also known as A + T-rich region or D-loop) [33, 61]. The J-strand generally contains 23 genes (nine PCGs, 14 tRNAs), and the N-strand includes 14 genes (four PCGs, eight tRNAs, and two rRNAs). There was an intergenic spacer or gene overlap between adjacent genes of the two mitogenomes, and no gene rearrangement was found. The number of intergenic spacers in the whole mitogenomes of *P*. *davidi* and *P*. *bimucronatus* was seven overlapping regions. The sum of their lengths was 34 bp and 36 bp, respectively. The intergenic spacers seem to mid-length in Coleoptera, for example, nearly twice that of the Tenebrionidae species [62] but shorter than *Coenochilus striatus* [45]. However, the longest intergenic spacers were both 19 bp and located between *trnS2* and *nad1*. There were 15 regions without intervals and overlaps. The number of overlapping region was 15, the sum of length was 40 bp, and the longest overlapping region was 8 bp, which was located between *trnC* and *trnW* (Fig 1 and Table 2), consistent with the results of some other Coleoptera species with only slight differences in length, such as the region of 9 bp and 8 bp of *Chrysochroa fulgidissima* and *Coomaniella copipes*, respectively [63, 64]. The gene arrangement, nucleotide composition, and codon usage may be used as a piece of very helpful information to assist the classification and infer evolutionary relationships.

**Table 2:** Organization of the mitogenomes of *Propomacrus davidi* and *P*. *bimucronatus*.

Gene	Direction	Position	Start codon	Stop codon	Anticodon	Intergenic
*trnI*	J	1–68 / 1–67			GAU / GAU	-3 / -3
*trnQ*	N	66–134 / 65–133			UUG / UUG	-1 / -1
*trnM*	J	134–202 / 133–201			CAU / CAU	0 / 0
*nad2*	J	203–1210 / 202–1209	ATT / ATT	TAA / TAA		5 / 5
*trnW*	J	1216–1283 / 1215–1282			UCA / UCA	-8 / -8
*trnC*	N	1276–1343 / 1275–1341			GCA / GCA	5 / 7
*trnY*	N	1349–1435 / 1349–1412			GUA / GUA	-2 / -2
*cox1*	J	1434–2967 / 1411–2944		T / T		0 / 0
*trnL2*	J	2968–3032 / 2945–3008			UAA / UAA	0 / 0
*cox2*	J	3033–3720 / 3009–3696	ATA / ATA	T / T		0 / 0
*trnK*	J	3721–3791 / 3697–3767			CUU / CUU	-1 / -1
*trnD*	J	3791–3854 / 3767–3830			GUC / GUC	0 / 0
*atp8*	J	3855–4010 / 3831–3986	ATT / ATT	TAA / TAA		-7 / -7
*atp6*	J	4004–4675 / 3980–4651	ATG / ATG	TAA / TAA		-1 / -1
*cox3*	J	4675–5461 / 4651–5437	ATG / ATG	TAA / TAA		0 / 0
*trnG*	J	5462–5525 / 5438–5502			UCC / UCC	0 / 0
*nad3*	J	5526–5879 / 5503–5856	ATT / ATT	TAA / TAA		-2 / -2
*trnA*	J	5878–5940 / 5855–5917			UGC / UGC	0 / 0
*trnR*	J	5941–6005 / 5918–5982			UCG / UCG	0 / 0
*trnN*	J	6006–6071 / 5983–6048			GUU / GUU	0 / 0
*trnS1*	J	6072–6139 / 6049–6116			UCU / UCU	0 / 0
*trnE*	J	6140–6202 / 6117–6179			UUC / UUC	-2 / -2
*trnF*	N	6201–6265 / 6178–6242			GAA / GAA	0 / 0
*nad5*	N	6266–7979 / 6243–7956	ATT / ATT	T / T		1 / 1
*trnH*	N	7981–8047 / 7958–8022			GUG / GUG	-1 / -1
*nad4*	N	8047–9384 / 8022–9359	ATG / ATG	TAA / TAA		-7 / -7
*nad4L*	N	9378–9668 / 9353–9643	ATG / ATG	TAA / TAA		2 / 2
*trnT*	J	9671–9734 / 9646–9709			UGU / UGU	-1 / -1
*trnP*	N	9734–9800 / 9709–9774			UGG / UGG	1 / 1
*nad6*	J	9802–10302 / 9776–10276	ATT / ATC	TAA / TAA		-1 / -1
*cytb*	J	10302–11444 / 10276–11418	ATG / ATG	TAG / TAG		-2 / -2
*trnS2*	J	11443–11507 / 11417–11482			UGA / UGA	19 / 19
*nad1*	N	11527–12477 / 11502–12452	ATT / ATT	TAA / TAA		1 / 1
*trnL1*	N	12479–12541 / 12454–12515			UAG / UAG	0 / 0
*rrnL*	N	12542–13835 / 12516–13808				0 / 0
*trnV*	N	13836–13904 / 13809–13877			UAC / UAC	-1 / -1
*rrnS*	N	13904–14688 / 13877–14661				0 / 0
A+T rich region		14689–18042 / 14662–18104				

Note: N and J indicate that the gene was located in the minor (N) and major (J) strand. The ‘/’ indicated that these from left to right were *P*. *davidi* and *P*. *bimucronatus*.

### Mitochondrial genome PCGs

**Fig 2:**
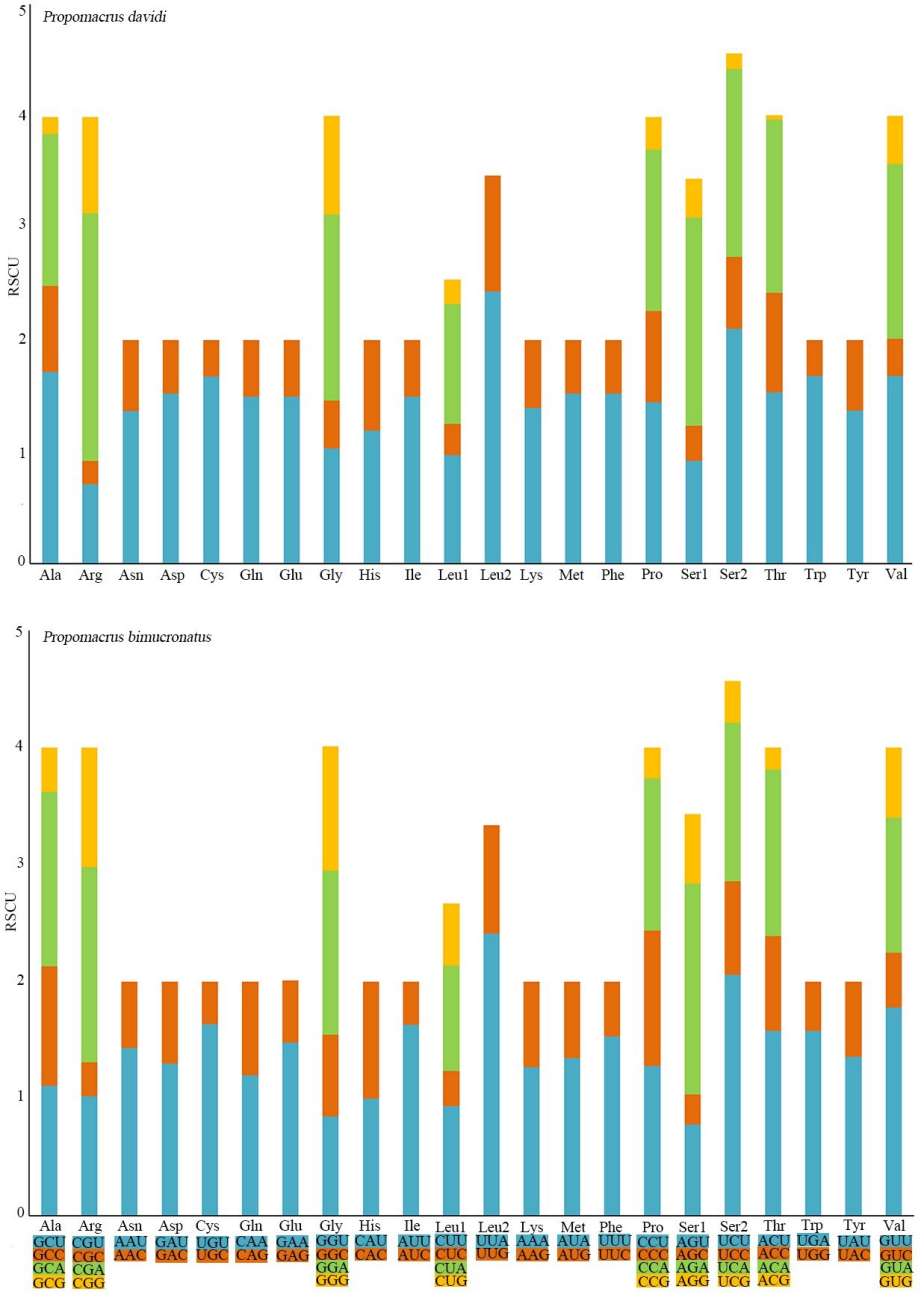
Relative synonymous codon usage (RSCU) in mitochondrial genomes of *Propomacrus davidi* and *P*. *bimucronatus*.

**Fig 3:**
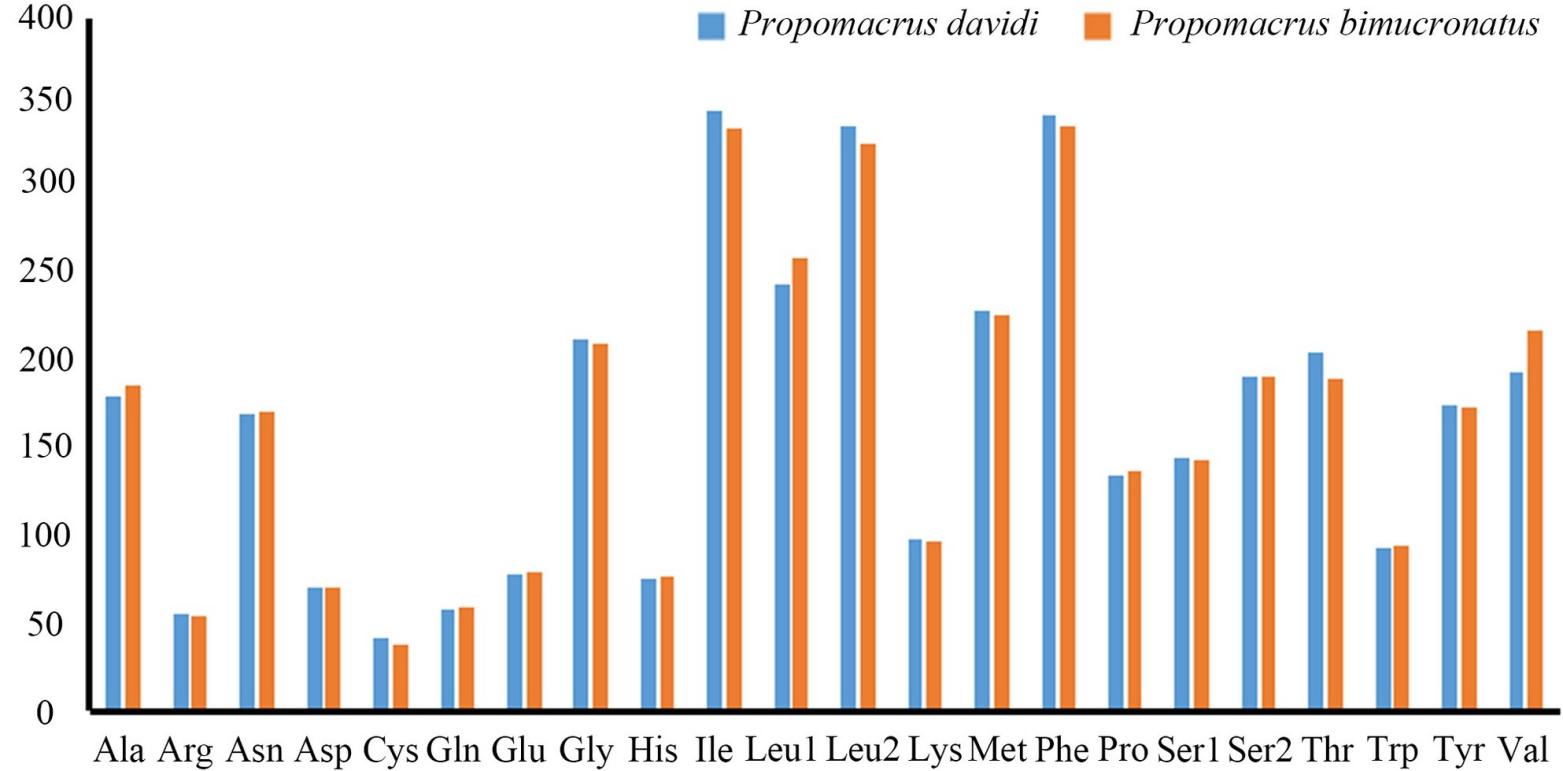
Codon distribution of *Propomacrus davidi* and *P*. *bimucronatus*. The codon families are represented on the X-axis.

The total length of the 13 PCGs was 11,137 bp for both *P*. *davidi* and *P*. *bimucronatus*, and the A + T content was 68.38% and 66.74%, respectively, these two species showed a negative AT-skew (−0.146, −0.163, respectively) and a negative GC-skew (−0.049 and −0.019, respectively) in PCGs (Table 3). The longest and shortest PCGs were *nad5* (1,714 bp) and *atp8* (156 bp). The combined 13 PCGs of *P*. *davidi* or *P*. *bimucronatus* mitogenomes encoded a total of 3,702 codons (excluding stop codons); the most frequently used codon was UUA (Leu2), and the relative synonymous codon usage (RSCU) was 2.43 and 2.41, respectively (Fig 2). The most frequently used amino acids were Ile, Phe, Leu1 and Leu2 (Fig 3).

**Table 3:** Base composition and skewness of *Propomacrus davidi* and *P*. *bimucronatus*.

Species	Items	Size(bp)	A%	T%	G%	C%	A+T%	G+C%	AT skew	GC skew
***Propomacrus davidi***	Genome	18042	39.06	30.43	9.94	20.57	69.49	30.51	0.124	-0.348
	PCGs	11137	29.19	39.19	15.03	16.58	68.38	31.61	-0.146	-0.049
	rRNA	2079	33.48	41.65	17.46	7.41	75.13	24.87	-0.109	0.404
	tRNAs	1478	37.69	36.27	14.55	11.50	73.96	26.05	0.019	0.117
	A+T region	3354	40.16	27.58	9.21	23.05	67.74	32.26	0.186	-0.429
***P*. *bimucronatus***	Genome	18104	36.74	31.16	11.96	20.13	67.91	32.09	0.082	-0.255
	PCGs	11137	27.92	38.82	16.31	16.95	66.74	33.26	-0.163	-0.019
	rRNA	2078	33.64	40.66	17.61	8.08	74.30	25.69	-0.094	0.371
	tRNAs	1450	38.28	36.07	13.93	11.72	74.35	25.65	0.030	0.086
	A+T region	3443	35.52	29.63	13.53	21.32	65.15	34.85	0.090	-0.224

Note: AT skew = (A − T)/ (A + T), GC skew = (G − C)/ (G + C).

The start codon of *cox1* was not detected in the two species, which may be related to the use of the non-canonical start codons of this gene [61, 65]; *nad6* genes were ATT and ATC, respectively; *nad2*, *atp8*, *nad3*, *nad5*, and *nad1* were typical ATT; *cox2* gene was ATA; *atp6*, *cox3*, *nad4*, *nad4L*, and *cytb* were typical ATG. The *cox1*, *cox2*, and *nad5* used the incomplete stop codon T, the complete stop codon of *cytb* was TAG, and the complete stop codons for other genes were the typical TAA (Table 2).

The start codons of *P*. *davidi* and *P*. *bimucronatus* differ from those of other Coleoptera species. In Coleoptera, except for the *cox1* gene, most protein-coding genes use a typical ATN as the start codon. However, incomplete stop codons (T or TA) are common in the mitochondrial genome of metazoans [66].

### The mitochondrial tRNA and rRNA genes

The total length of 22 tRNA genes in the mitochondrial genomes of *P*. *davidi* and *P*. *bimucronatus* was 1,478 bp and 1,450 bp, respectively. Most tRNAs exhibit the typical cloverleaf structures, except trnS1 was missing the DHU arm and the anticodon was changed from GCU to UCU. Although most arthropods use a GCU anticodon in tRNA-Ser(AGN), almost all beetle mitogenomes published so far have the UCU anticodon for this tRNA [61]. From the known complete mitogenome of Coleoptera insects, Polyphage uses UCU as an anticodon, and the other suborders of Archostemata, Myxophaga, and Adiphaga are GCU [66]. This suggests that the anticodon may be a molecular synapomorphy of Coleoptera [61, 66].

**Fig 4:**
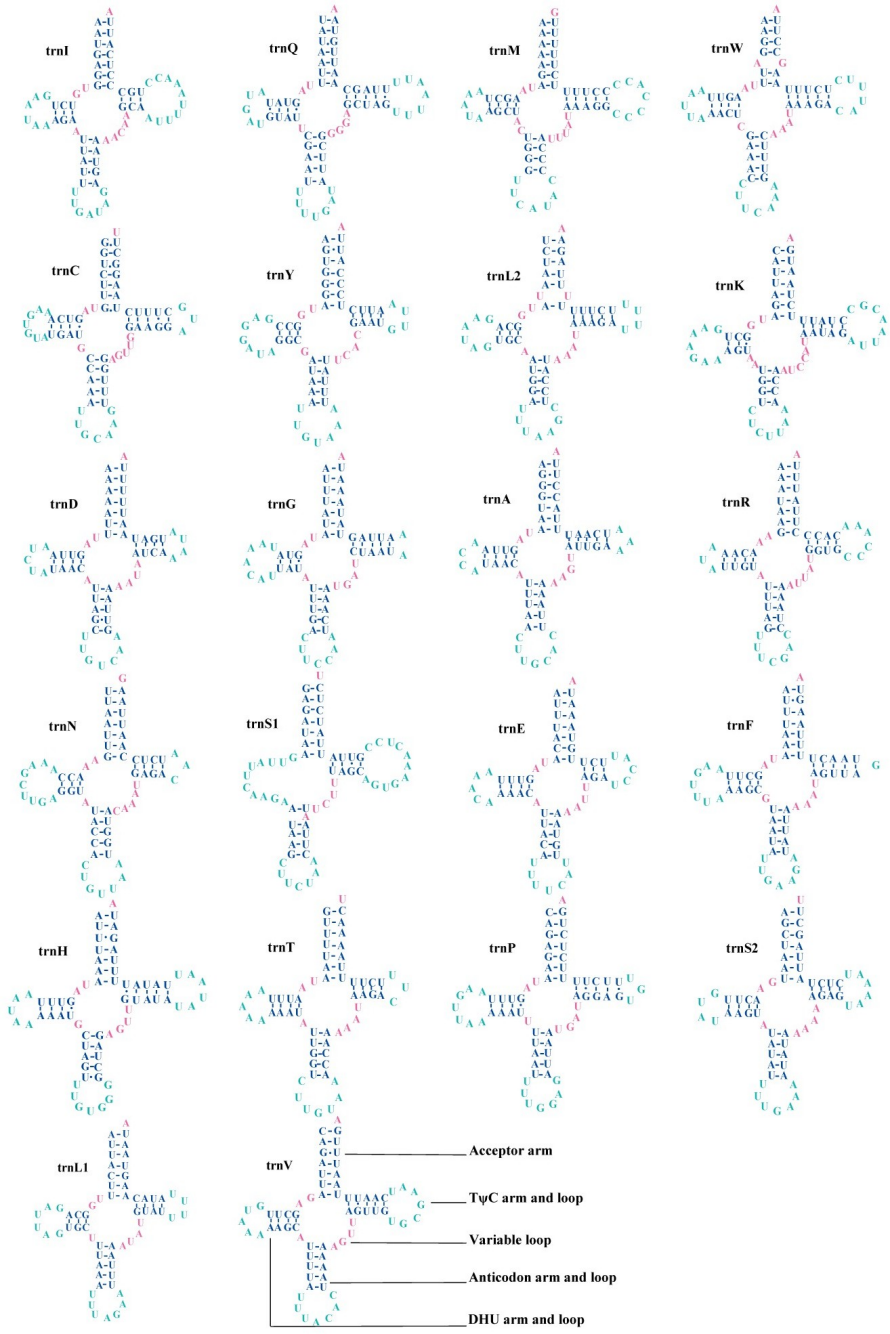
Secondary structure of tRNA in mitochondrial genes of *Propomacrus davidi*.

**Fig 5:**
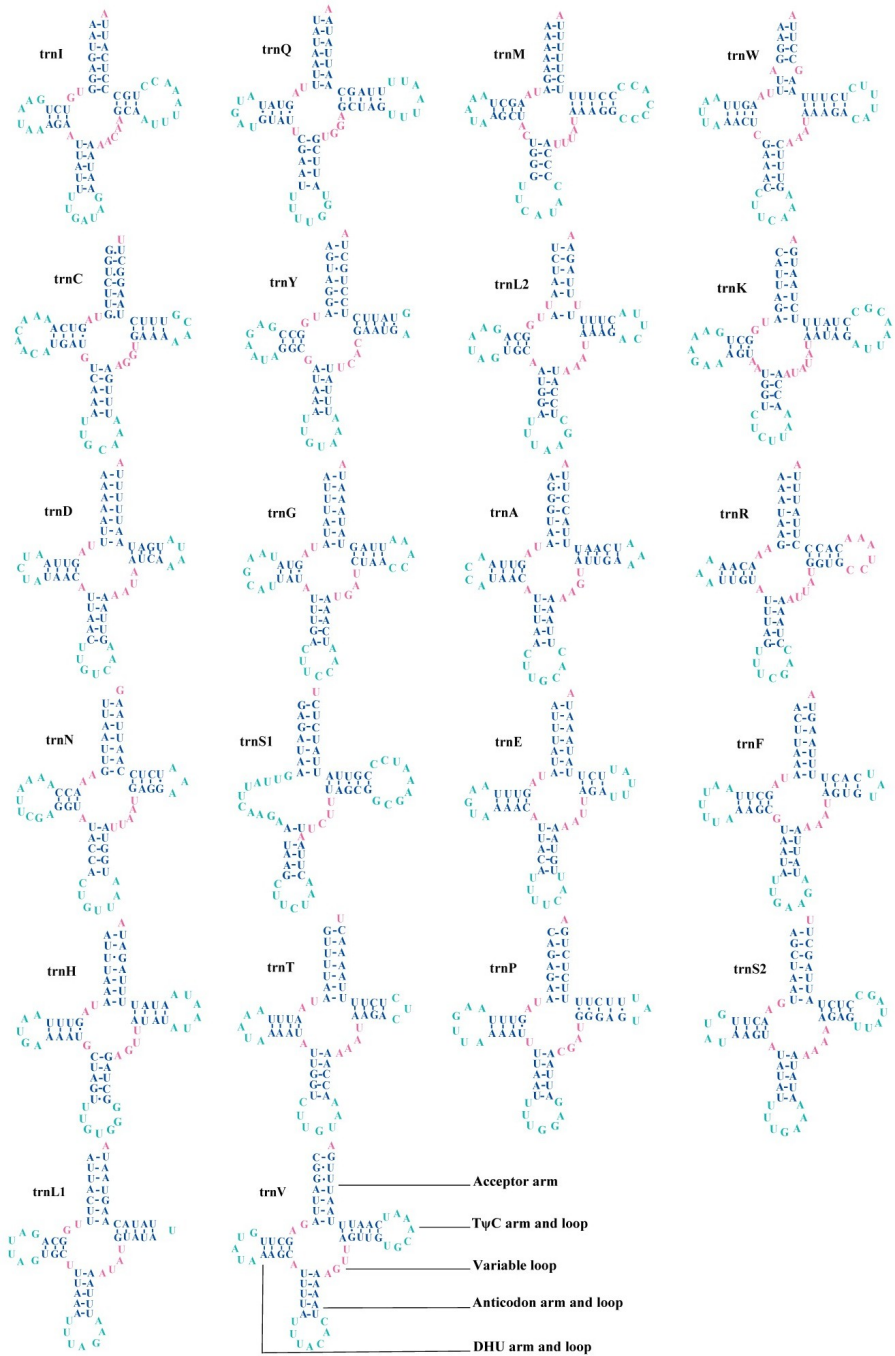
Secondary structure of tRNA in mitochondrial genes of the *Propomacrus bimucronatus*.

In the folding process of the cloverleaf structure, there were 26 pairs of G-U mismatches in *P*. *davidi* (Fig 4), of which nine pairs were located in the amino acid acceptor arms of *trnQ*, *trnC* (3), *trnY*, *trnA*, *trnF*, *trnH* and *trnV*; seven pairs were located in the DHU arms of *trnQ* (2), *trnC*, *trnK*, *trnG*, *trnH* and *trnP*, and seven pairs were located in the TΨC arms of *trnQ*, *trnC*, *trnS1*, *trnH*, *trnP* (2), and *trnV*; three pairs of anticodon arms were located at *trnI*, *trnD*, and *trnH*. There were 24 pairs of G-U mismatches in *P*. *bimucronatus* (Fig 5), of which eight pairs were located on the amino acid acceptor arm of *trnC* (3), *trnY*, *trnA*, *trnH*, and *trnV* (2); seven pairs were located on the DHU arm of *trnQ* (2), *trnC*, *trnK*, *trnG*, *trnH*, and *trnP*; eight pairs were located on the TΨC arm of *trnQ*, *trnY*, *trnN*, *trnS1*, *trnP* (3), and *trnV*, and one pair was located on the anticodon arm of *trnH*. The unpaired base A appeared in the anticodon arm of *trnS1* in the two species.

**Fig 6:**
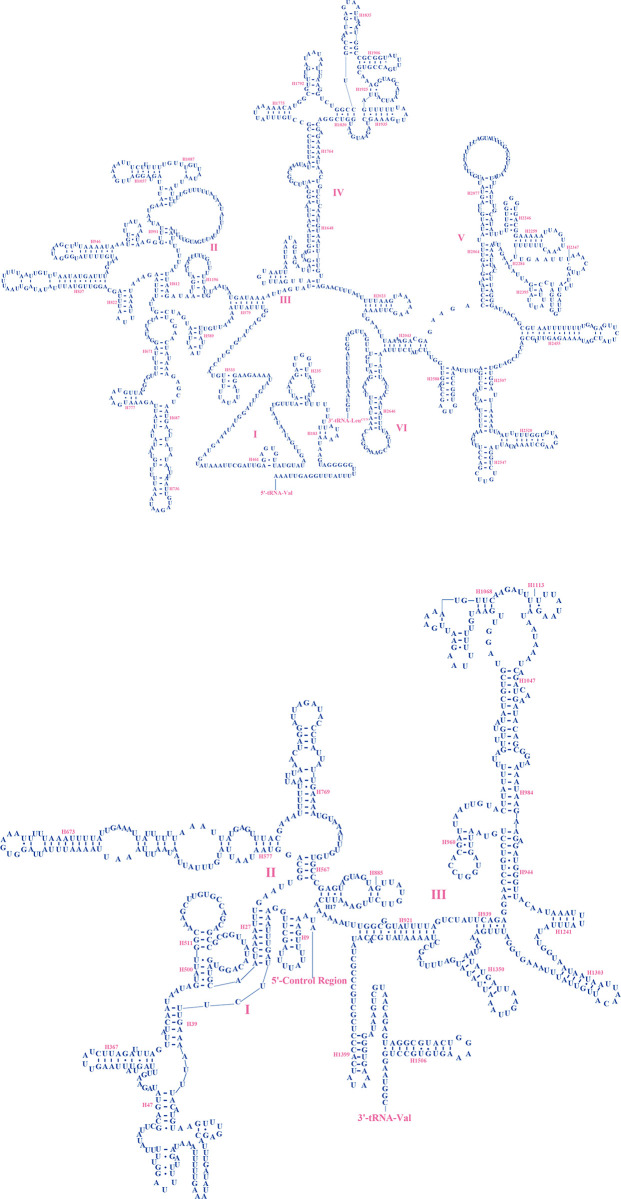
a. Predicted secondary structure of the *rrnL* gene in *Propomacrus davidi*. The inferred Watson Crick bonds are represented by lines, while the GU bond is represented by dots. b. Predicted secondary structure of the *rrnS* gene in *Propomacrus davidi*. The inferred Watson Crick bonds are represented by lines, while the GU bond is represented by dots.

**Fig 7:**
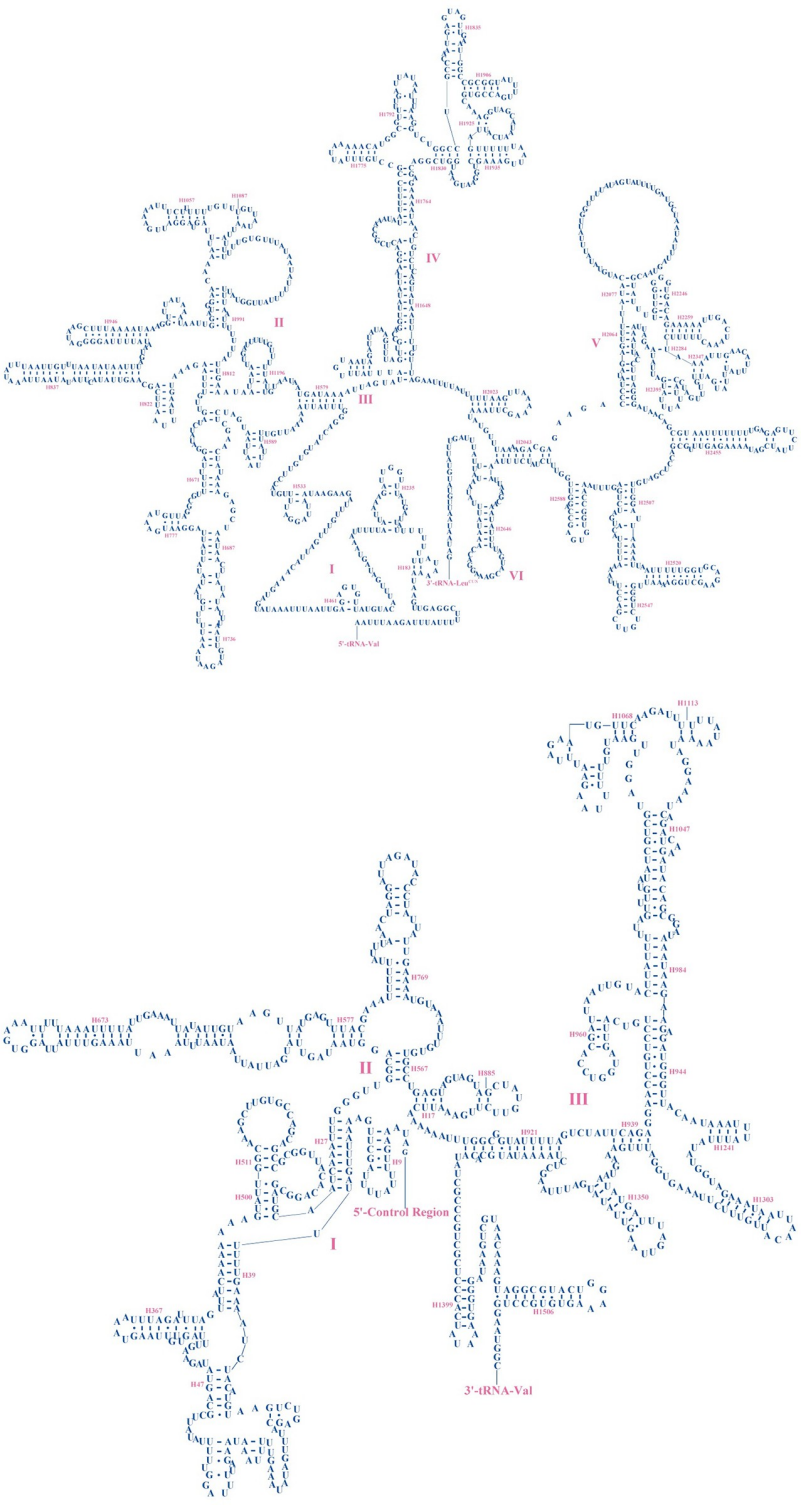
a. Predicted secondary structure of the *rrnL* gene in *Propomacrus bimucronatus*. The inferred Watson Crick bonds are represented by lines, while the GU bond is represented by dots. b. Predicted secondary structure of the *rrnS* gene in the *Propomacrus bimucronatus*. The inferred Watson Crick bonds are represented by lines, while the GU bond is represented by dots.

*rrnL* genes of the two beetles were located between the *trnL1* and *trnV*, and *rrnS* genes were located between *trnV* and control region, as in other insects [31, 34, 61], and *rrnL* had greater variation than *rrnS*. The sequence lengths of the *rrnL* (Fig 6A) and *rrnS* (Fig 6B) of *P*. *davidi* were 1,294 bp and 785 bp, respectively, and the A + T contents were 76.43% and 72.99%, respectively. The sequence lengths of the *rrnL* (Fig 7A) and *rrnS* (Fig 7B) of *P*. *bimucronatus* were 1,293 bp and 785 bp, respectively, and the A + T contents were 76.1% and 71.34%. The secondary structures of *rrnL* in the two species were predicted to have 42 helical structures and five domains (I, II, IV, V, VI). The domain III deletion of *rrnL* is a typical feature of arthropods [31, 34, 67–69]. The secondary structures of *rrnS* have 26 helical structures and three domains.

### Control region

**Fig 8:**
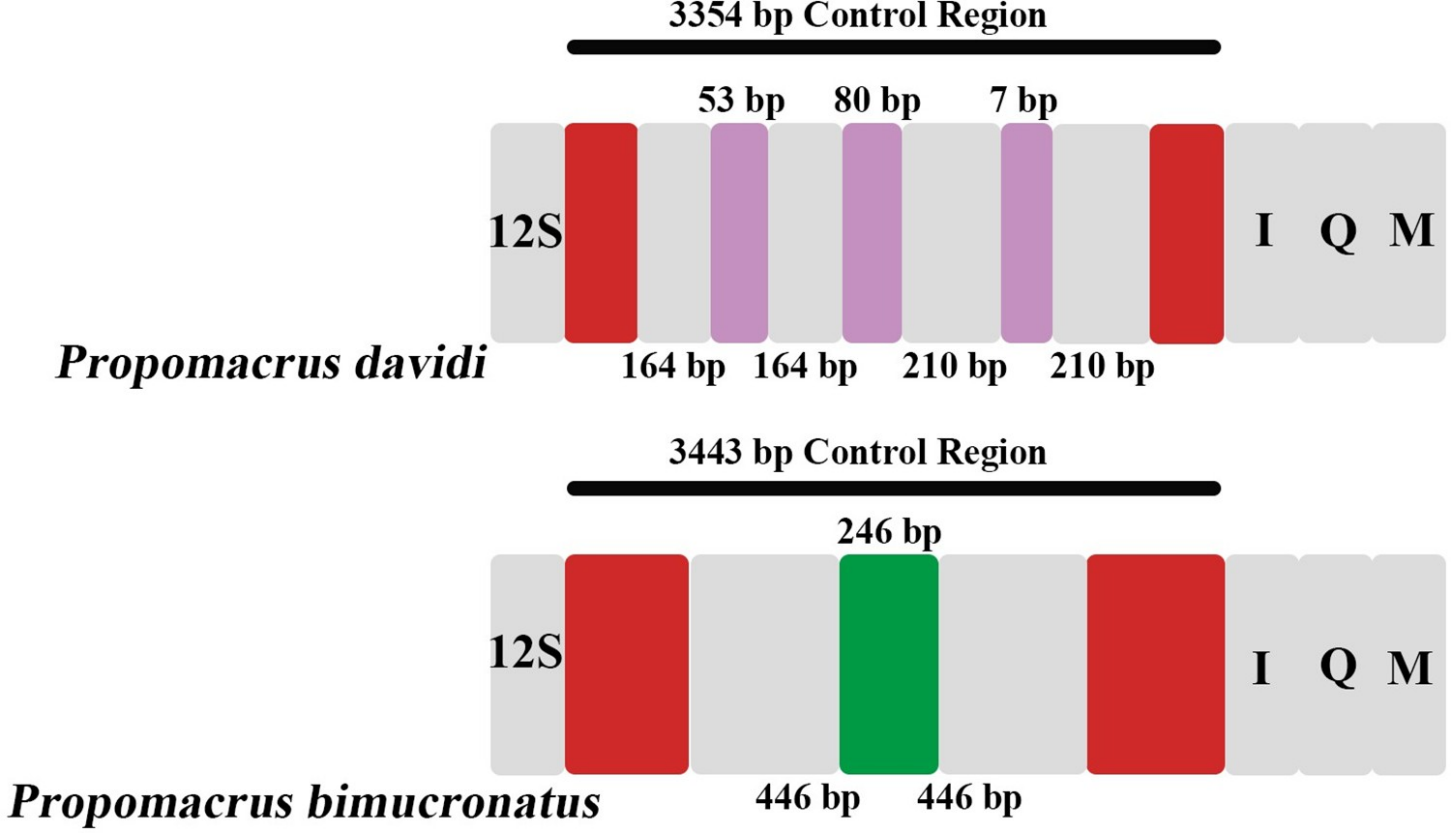
The control region of mitochondrial genome of *Propomacrus davidi* and *P*. *bimucronatus*. Non-repetitive unit (red), overlapping regions (green), intergenic spacer (purple), repeat unit (grey).

The mitogenome control region is a non-coding region of the sequence in its genome, also known as the A+T-rich region, which might be the key to characterizing typical CRs in mitogenomes [70, 71]. The control region is thought to regulate the replication and transcription of the mitogenome [72, 73]. The total lengths of the A+T-rich region of *P*. *davidi* and *P*. *bimucronatus* mitogenomes were 3,354 bp and 3,443 bp, respectively, both located between *rrnS* and *trnI*. However, the difference between these two species in CRs was not limited by these features and varied considerably. The T content in the control region is always higher than that in the coding region, which is a well-documented pattern in the insect mitogenomes [74]. The A + T contents were 67.74% for *P*. *davidi* and 65.15% for *P*. *bimucronatus*; these two species showed a positive AT-skew (0.186 and 0.090, respectively) and a negative GC-skew (−0.429 and −0.224, respectively). There were two pairs of repeat units in the 15,475–16,362 bp region in the mitogenome of *P*. *davidi*. The interval between the two pairs of repeating units was 80 bp, and the repeat sequence length for *P*. *davidi* and *P*. *bimucronatus* was 164 bp and 210 bp, respectively. The interval between the 164 bp repeat sequences was 53 bp, and the interval between the 210 bp repeat sequences was 7 bp. In the *P*. *bimucronatus* mitogenome, there was a pair of repeat units with a length of 446 bp at 15, 504–16,149 bp region, and the length of the overlapping region between the repeat sequences was 246 bp (Fig 8). The control region of the two species also has one poly-T with a length of 20 bp. There were many microsatellite structures in the control region. *P*. *davidi* had a total of 12 (TA)_n_ structures, including one (TA)_8_, three (TA)_5_, one (TA)_7_, four (TA)_3_, two (TA) _4_ and one (TA)_10_. *P*. *bimucronatus* has nine (TA)_n_ structures, one (TA)_8_, one (TA)_7_, and seven (TA)_3_.

### Phylogenetic analysis

**Fig 9:**
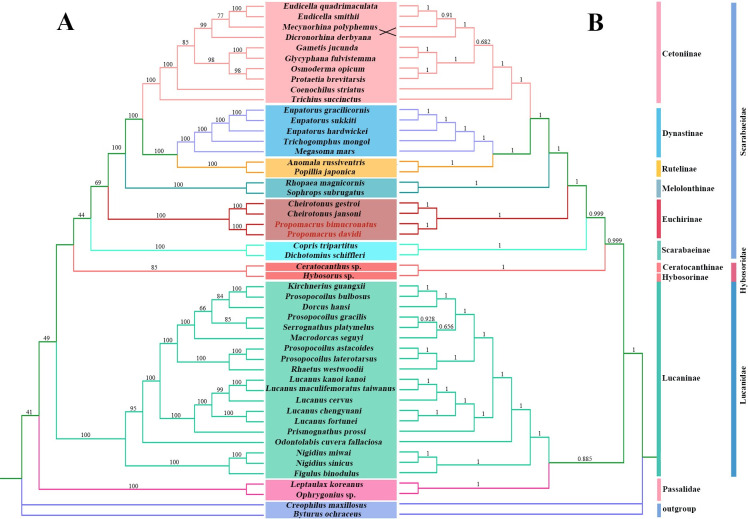
Phylogenetic tree of Scarabaeoidea produced from maximum likelihood (ML) (A) and Bayesian inference (BI) (B) based on 13 PCGs. Bayesian posterior probabilities are indicated on branches. The two species sequenced in this study are highlighted in red.

Based on the 13 PCG datasets of 50 species, ML and BI trees with almost the identical topology were generated (Fig 9). The difference between the two trees is only the position of *Mecynorhina polyphemus* and *Dicronorhina derbyana*, and the two species have only a slight exchange of positions. The phylogenetic relationships at the subfamily level of Scarabaeidae constructed by the study are: ((((Cetoniinae + (Dynastinae + Rutelinae)) + Melolonthinae) + Euchirinae) + Scarabaeinae. Among the 25 species of Scarabaeidae, the four species of Euchirinae (*P*. *davidi*, *P*. *bimucronatus*, *C*. *jansoni*, *C*. *gestroi*) clustered into the same branch in the phylogenetic tree and were the most closely related, showing Euchirinae monophyly and a nested position within the Scarabaeidae, supporting the morphological characteristics conclusion of Šípek et al. (2011). At the same time, the results continue to support the phylogenetic relationship among the subfamilies Cetoniinae, Dynastinae, Rutelinae, Melolonthinae, and Euchirinae [6, 16, 29, 31, 75, 76]. In addition, the unclear phylogenetic relationships at the genus level also indicate to us that the data are insufficient. We need more basic research to support the species boundaries and evolutionary relationships of this charismatic-looking beetle.

The superfamily Scarabaeoidea constructed by ML (Fig 9A) and BI (Fig 9B) methods are: ((Scarabaeidae + Hybosoridae) + Lucanidae) + Passalidae. Scarabaeidae and Hybosoridae are sister groups, and the formation of a clade between the two families is supported strongly. Although it is different from the phylogenetic relationship of the families of Scarabaeoidea based on studies from nuclear protein-coding genes [77, 78], which indicated that the Lucanidae did not form a sister-group relationship with the Scarabaeidae and Hybosoridae, the results support the monophyly of the four families and are consistent with results of morphological characteristics studies [17, 18].

## Conclusion

In this study, the complete mitogenome sequences of *P*. *davidi* and *P*. *bimucronatus* were sequenced and analyzed; the total lengths of the two species were 18,042 bp and 18,104 bp, respectively, which are longer than those of most metazoans, and the genomes showed an obvious AT-skew. Each gene composition and sequence were consistent with those of ancestral insects, including 13 PCGs, 22 tRNAs, two rRNAs, a control region, and no gene rearrangements or deletions. The mitogenome of the two species uses UCU as an anticodon. The secondary structure models of *rrnL* and *rrnS* were similar to those of other insect genes, and *rrnL* had greater variation than *rrnS*. The domain III deletion of *rrnL* is a typical feature of arthropods. The T content in the control region is always higher than in the coding region, a well-documented pattern in the insect mitogenome. The total lengths of the A+T-rich region of *P*. *davidi* and *P*. *bimucronatus* mitogenomes were 3,354 bp and 3,443 bp, respectively, both located between *rrnS* and *trnI*.

The analysis based on the ML and BI method of 13 PCGs produced a well-resolved framework supporting the conclusion that Euchirinae is a monophyletic subfamily of Scarabaeidae. Mitogenome analysis is useful for comparing genetic relationships at classificatory levels. More research is needed to reconstruct comprehensive phylogenies to better resolve the phylogenetic relationships of Euchirinae.
